# Gaussian Process Regression Plus Method for Localization Reliability Improvement

**DOI:** 10.3390/s16081193

**Published:** 2016-07-29

**Authors:** Kehan Liu, Zhaopeng Meng, Chung-Ming Own

**Affiliations:** 1 School of Computer Software, Tianjin University, Tianjin 300350, China; kehanliu@tju.edu.cn; 2 School of Computer Software, Tianjin University of Traditional Chinese Medicine, Tianjin 300193, China; mengzp@tju.edu.cn

**Keywords:** location estimation, RSS fingerprinting, Gaussian Process Regression, Naive Bayes

## Abstract

Location data are among the most widely used context data in context-aware and ubiquitous computing applications. Many systems with distinct deployment costs and positioning accuracies have been developed over the past decade for indoor positioning. The most useful method is focused on the received signal strength and provides a set of signal transmission access points. However, compiling a manual measuring Received Signal Strength (RSS) fingerprint database involves high costs and thus is impractical in an online prediction environment. The system used in this study relied on the Gaussian process method, which is a nonparametric model that can be characterized completely by using the mean function and the covariance matrix. In addition, the Naive Bayes method was used to verify and simplify the computation of precise predictions. The authors conducted several experiments on simulated and real environments at Tianjin University. The experiments examined distinct data size, different kernels, and accuracy. The results showed that the proposed method not only can retain positioning accuracy but also can save computation time in location predictions.

## 1. Introduction

Accurate, reliable, and real-time indoor positioning and position-based protocols and services are required in future social applications. Through a mobile device, a positioning system can help to determine its position and makes the position of the device available for position-based services such as navigation and tracking or monitoring. The location information of devices or users could considerably improve the performance of wireless network for network planning, network adaptation, and load balancing. Generally, the indoor localization problem is resolved using standard, low-cost, and already deployed infrastructure such as the strength database of received signals [1,2]. Instead of spending resources in deploying dedicated infrastructure and collecting data procedure, the purpose of indoor positioning study is to design and implement data fusion methods and algorithms using existing infrastructure.

Generally, received signal strength (RSS)-based location fingerprinting is based on the principle that each position has a unique set of signal values. When a system boots up, mobile devices receive the unique RSS value of the system, and they search the fingerprinting database and identify the entry that is most similar to the system unique RSS value as the estimated location. The main problem of a typical RSS fingerprinting system is that the real RSS value at any location is easily affected by the object (human or furniture inside the building) and multipath fading effects [3]. In other words, the RSS fingerprint obtained at different periods of time need not match the previous fingerprint stored in the database, leading to incorrect estimation results. Previous researchers have shown that the fingerprinting localization method can achieve meter-level accuracy if the RSS signature map is not outdated. However, because of the dynamic nature of the radio channel and changes in the surrounding environment, the RSS signature maps must be updated multiple times daily. That is, the time and effort required to build the RSS signature map during the offline phase are the major drawbacks of fingerprinting-based localization. 

In addition, the challenges in deploying reliable and scalable fingerprinting localization systems and facing user limits are also the barriers to reaching the accuracy required for use in commercial applications. Many studies have prompted in the elimination of the times at gathering RSS signatures. In [4], Haeberlen et al. demonstrated a system that allows for remarkably accurate localization across an entire office building over 12,000 square meters in area; most notably, regarding the scale, they localized a device to one of 510 cells in the building within seconds, yielding a success rate of greater than 95%. Moreover, Bisio et al. designed an indoor localization scheme of targets by using electromagnetic waves; this system was replaced by an offline phase in which the fingerprints in each point of the area of interest were estimated by means of electromagnetic waves [5]. In [6], the authors computed a position by considering the RSS measurements as a part of a random process, exploiting the information present in the acquired signals. With the efforts to build an RSS signature map having been reduced, the median error remains unacceptable for most practical indoor applications [7]. Furthermore, Zheng et al. utilized both the raw RSS values and their relation to construct a new stable and robust fingerprint for indoor position, their results indicated that the RSS variance problem can be solved without any manual calibration [8]. In this study, we developed a probabilistic framework for handling sparse training data in fingerprinting localization. Specifically, the calibration effort and costs of building the RSS signature map were reduced by modeling signal strength by using the advanced Gaussian process (GP), and the troublesome of RSS signature map was enhanced in the online phase.

Supervised learning in the form of regression and classification is an important constituent of statistics and machine learning, either for the analysis of data sets or as a subgoal of a more complex problem [9]. Traditionally, parametric models have been used for this purpose. A possible advantage also exists in the ease of interpretability; however, for complex data sets, simple parametric models may lack expressive power. A GP is a nonparametric model that is characterized completely by its mean function and covariance matrix [10]. A GP depends on several hyper parameters that can be estimated using training measurements. In our study, we used the properties of the conditional probability of a GP. The GP prior distribution was used for regression and predicting the RSS at locations with no prior measurements, and the Naive Bayes algorithm was also used to derive the obtained conditional Gaussian probability. We named the computation process Gaussian Process Plus. A major advantage of using a GP-based predicator is that in addition to the mean estimate of the RSS, an estimation of the variance is also produced, providing an indication of the uncertainty of the estimation [11]. The aforementioned process can be performed offline and generally must be performed only once.

Yiu et al. used Gaussian process regression (GPR) for received signal strength indication (RSSI) prediction in order to solve indoor location problems [12]. Partial measurements were first taken from the area of interest. They then used the Firefly algorithm to train and categorize the prior results derived using GPR. The trained results were used to build a refined fingerprinting database for the entire area of interest. The experiment showed that the GPR model could achieve a satisfactory result in predicting the RSS of an area with no prior measures. However, according to Yiu et al., the ability to expand the GPR model is restricted by the need to rebuild the fingerprinting database; in addition, the flexibility in the computation of the GPR models was limited in their study. Thus, to retain the flexibility of GP and increase the efficiency of indoor positioning, we proposed the Gaussian Process Regression Plus (GPRP) method to solve the aforementioned limitations, and we tested the experiments in both simulated and real environments. 

The remainder of this manuscript is organized as follows: Section 2 reviews related studies investigating the properties of RSSI for signal transmission, and the definitions of GP. Section 3 details the GPRP measurement system used in the current study and describes the data analysis method. Section 4 presents the simulated and real experiments and discussion, and Section 5 offers conclusions and recommendations for future research.

## 2. Preliminaries

### 2.1. RSS Fingerprint

RSS properties, which facilitate location fingerprinting, have been determined by many studies examining indoor positioning systems. This research demonstrates that user orientation could cause a variation of up to 5 dBm in the RSSI level [13,14]. At any location, the different orientations of users and mobile devices with respect to the transmitter could cause the mean RSS value to change. The modeling of the RSS-based location fingerprinting is essential for location determination algorithms; examples of RSS-based location fingerprinting models are the probabilistic approach model and preliminary analytical model [15]. Gaussian or lognormal distributions were used to model the randomness of RSS. For example, [16] summarized in a large-scale measurement that most RSS histograms could be fitted appropriately with Gaussian distributions but that few histograms could be fitted with bimodal Gaussian distributions.

**Figure 1:**
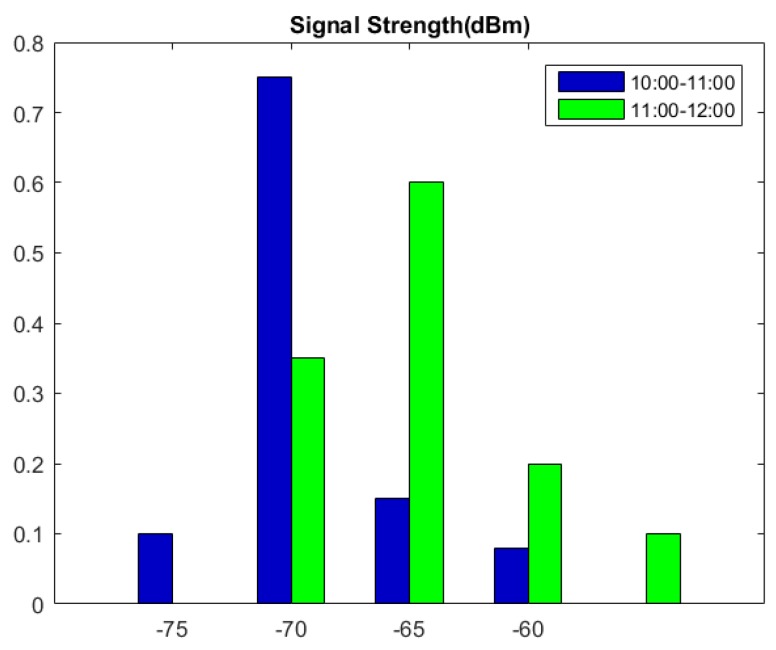
RSS distributions during different time periods in the cafeteria.

Current signal-based RSS location systems have two problems. First, a considerable manual calibration effort is required to construct a radio map in the offline training phase; second, the positioning accuracy changes with the environmental dynamics. Three dynamic factors observed to change frequently over time in the environment are proposed in [17], including the presence of people, relative humidity, and movement. These factors easily affect the radio signal propagating from access points (APs) to mobile devices and are responsible for changes in positioning accuracy. The results of the experiments conducted in a cafeteria showed that an RSS distribution at a fixed location with a specified beacons was not constant with time (Figure 1). The RSS values calibrated previously in the radio map may be outdated; this condition degrades positioning accuracy because of the presence of people.

Traditionally, an average RSS has been considered to be lognormally distributed according to a large-scale fading model. Such RSS is generally predictable and follows several standardized path loss models.

The wall attenuation factor (WAF) model is useful for describing the slow-fading phenomenon and attenuation in signal propagation in indoor environments [18]. In this model, the attenuation factor is used to predict signal propagation behavior when walls are the main obstacle. The following equation shows how attenuation influences RSS:

(1) $$P{\left(d\right)}_{dbm}=P{\left(d_{o}\right)}_{dbm}-10\times n\times \log\left(\frac{d}{d_{o}}\right)-nW\times WAF$$

where *n* indicates the rate of increase in signal attenuation with the propagation distance, $P\left(d_{o}\right)$ is the RSS at a distance of reference point $d_{o}$, and d is the distance between the transmitter and the receiver. Furthermore, nW is the number of obstacles (walls) between the transmitter and the receiver, and *WAF* is the attenuation value resulting from the obstacles. In this equation, if nW is greater than a certain constant *C*, the value of nW is considered to represent the number of walls at which the attenuation factor stops influencing the signal. We can then use the constant value instead of nW. When we select a subset of APs satisfying a certain property in our alternative set of fingerprint definitions, we retrieve the corresponding position from the offline fingerprinting database by using the location estimation algorithm. Generally, several methods are used for determining the nearest neighbor location. For example, we can use the Euclidean distance:

$$\text{Eucdist}\left(S,R\right)=\sqrt{\sum_{i=1}^{n}{\left(s-r_{i}\right)}^{2}}$$

or the Mahalanobis distance:

$$\text{Mahaldist}\left(S,R\right)=\sqrt{{\left(S-R\right)}^{T}S^{-1}\left(S-R\right)}$$

where *S* is the RSS value for the target location and *R* is the value closest to *S* in the fingerprinting database. 

### 2.2. Gaussian Process

The GPR is a new machine-learning method based on Bayesian theory and statistical learning theory. It provides a flexible framework for probabilistic regression and is widely used to solve high-dimension, small-sample, or nonlinear regression problems. From the view of the function space, the GP defines a distribution over functions. GPs are the extension of multivariate Gaussian model to the infinite-sized vector of real-valued variables. The GP is fully specified by a mean function and a covariance function such as:

(2) $$\{\begin{array}{c} m\left(x\right)=E\left[f\left(x\right)\right]\\ k\left(x|x^{\prime }\right)=E[\left(f\left(x\right)-m\left(x\right)\right)\left(f\left(x^{\prime }\right)-m\left(x^{\prime }\right)\right)] \end{array}$$

where $x\text{ and }x^{\prime }\epsilon R$ are random variables [19]. The GP equation is $f\left(x\right)\sim gp\left(m\left(x\right),k\left(x,x^{\prime }\right)\right)$. For simplification, the mean function is usually set to zero in the data preprocessing stage. 

Because the key assumption in GP modeling is that the data can be represented as a sample from a multivariate Gaussian distribution, we can infer that:

(3) $$\left[\begin{array}{c} x\\ x^{*} \end{array}\right]\sim N\left(0,\left[\begin{array}{cc} K & K_{*}^{T}\\ K_{*} & K_{**} \end{array}\right]\right)$$

where T indicates matrix transposition. The function probability follows a Gaussian distribution, and the conditional probability of $x_{*}|x$ can be computed as:

(4) $$p\left(x_{*}|x\right)\sim N\left(K_{*}K^{-1}y,K_{**}K^{-1}K_{*}^{T}\right)$$

The optimal estimation for $x_{*}$ is the mean of this distribution $\bar{x_{*}}=K_{*}K^{-1}x$ [20,21], and the uncertainty in the estimation is the variance $\text{var}\left(x_{*}\right)=K_{**}-K_{*}K^{-1}K_{*}^{T}$. The aforementioned description indicates that the GPR is suitable for predicting unknown data with limited known data. GPR is also the central idea used in this study.

Some works have used the GP to help generate a fingerprint database in the offline phase [22,23]. Ferris et al. demonstrated how to use the GP to generate likelihoods at locations for which no calibration data were available, and proved that Gaussian regression could be applied successfully to various localization problems [22]. Atia et al. [24]. considered using the GPR technique to bridge the GPS outage; they ultimately obtained an 80% improvement in the position root square mean error (RMSE). Richter et al. analyzed different GPR models for WLAN fingerprinting and provided useful advice regarding GPR [22]. Cho et al. created a GPR-based radio map construction method for surveying data that could obtain high accuracy and availability, though realistic data were rare [25]. These studies have proved that GPR is a viable means of improving positioning accuracy and releasing the labor at collecting fingerprinting data.

### 2.3. Weight-RSS Propagation Model

For estimating and tracking the parameters of RSS propagation model in the indoor environment, many studies employ adaptive Bayesian framework to cope with unpredictable, inter-calibration and fading radio effects. However, these methods cannot drive the acceptable performance due to the complex propagation phenomena in the radio transmission. In [8], Zheng et al. proposed weight-RSS, a calibration-free solution to solve the RSS variance caused by device heterogeneity and complex environmental factors. In Zheng’s system, their location can be estimated by matching all the RSS values and the relation from the test device with all the entries of the fingerprint mapping results.

Assume that *m* APs are deployed in an indoor environment and the physical space of the indoor environment is modeled as a finite space *L* = {$l_{1}\left(x_{1},y_{1}\right),\;l_{2}\left(x_{2},y_{2}\right),\;\ldots ,\;l_{n}\left(x_{n},y_{n}\right)\}$ [8]. RSS values of all APs at the location $l_{i}$ can be represented as follows:

$$R_{i}=\left(r_{i}^{1},\;r_{i}^{2},\;\ldots ,\;r_{i}^{q},\ldots ,\;r_{i}^{m}\right)$$

and the weight-RSS of location $l_{i}$ is defined as:

$$D_{j}=\left\{\left(r_{i}^{1},s\left(r_{i}^{1}\right)\right),\;\left(r_{i}^{2},s\left(r_{i}^{2}\right)\right),\;\ldots ,\left(r_{i}^{m},s\left(r_{i}^{m}\right)\right)\right\}$$

where $s\left(r_{i}^{j}\right)$ is the index of $r_{i}^{j}\;$after $R_{i}$ was sorted as descending order. Accordingly, Zheng et al. assume that the distance from the fingerprint map is $D_{\text{tr}}$ (off line), and the distance from a test device is $D_{\text{te}}$ (on line). The weighting factor is computed as follows:

$$F_{\text{tr},\text{te}}=\{f_{i}|\;1\leq i\leq m\}$$

and:

$$f_{i}=1-\frac{\left|s\left(r_{\text{tr}}^{j}\right)-s\left(r_{\text{te}}^{j}\right)\right|}{\text{max}\left(s\left(r_{\text{tr}}^{j}\right)-s\left(r_{\text{te}}^{j}\right)\right)}$$

Then the authors can derive the difference between distance from fingerprint map $D_{\text{tr}}$ and from a test device $D_{\text{te}}$ is:

$$Dist\left(D_{\text{tr}},\;D_{\text{te}}\right)=\sqrt{\sum_{j}^{m}f_{i}{\left(r_{\text{tr}}^{j}-r_{\text{te}}^{j}\right)}^{2}}$$

## 3. System Design

In [12], Yiu et al. used the trained GPR model to estimate the signature map of an area. For optimizing hyper parameters in GPR, they proposed that the signature map be rebuilt; however, inefficient computation restricted the flexibility of the Gaussian model. Thus, in our study, we focused on GPR system improvement and used the Naive Bayes algorithm to reduce the computation complexity of the indoor positioning system, and retain the flexibility of the models of GP.

**Figure 2:**
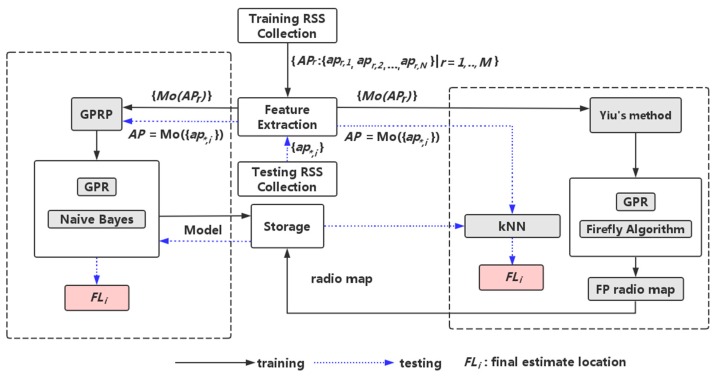
Framework of GPRP’s method compared to Yiu’s method.

In Figure 2, we illustrate the proposed system framework and compares it with that of Yiu et al. Firstly, our GPRP method and Yiu’s method created a discrete and partial RSS fingerprinting database in the testing area. Accordingly, we all derived and tested several GP models for system computation. The different GP models had distinct distribution functions and hyper parameters. However, Yiu et al. proposed a method based on categorized and GP algorithms, and finished with the rebuilding of the RSS fingerprinting database; by contrast, our GPRP method employed the Naive Bayes method to select all the predicted location from the GP models, and the computation was more easily derived and was less time-consuming. Furthermore, to stress the efficiency of our proposed method, in Figure 2, the solid line indicates the training phase, and the blue dashed line indicates the testing phase. $FL_{i}$ was derived as the final estimated location for each method. The less computation steps are needed in our GPRP method.

### 3.1. The Method of Gaussian Process Regression Plus

In our method, an offline RSS fingerprinting database must be built first. We assume that the testing area has *M* reference points in locations $L_{r}\left(r\in \left\{1,2,\cdots ,M\right\}\right)$. In the sampling phase, we collect the RSS value at the *r*th reference point of N APs from 1 to *j*th times; that is:

$$AP_{r}=\left\{ap_{r,1},ap_{r,2},\cdots ,ap_{r,N}\right\}$$

where:

$$ap_{r,1}=Mo\left(\left\{ap_{r,1}^{1},\;ap_{r,1}^{2},\;\ldots ,ap_{r,1}^{j}\right\}\right)$$

*Mo*(.) is the mod function, which is used to derive the remainder of the division. $ap_{r,N}^{j}$ is denoted as the odd moment data at the *r*th reference point of N APs from 1 to *j*th times; by contrast, $AP_{r}$ is the filtering set of signal strength received at the *r*th reference point.

When we attempt to predict the unknown position at the input of RSS values, we obtain a series of RSS values $AP_{*}$ as follows:

$$AP_{*}=\left\{ap_{*,1},ap_{*,2},\cdots ,ap_{*,N}\right\}$$

Because of the noises in our environment, we can coincide with the regression problem as $L\left(AP_{*}\right)=f\left(AP_{*}\right)+\nu$ fort the simplification. The latent function f(.) describes the relationship between the RSS and spatial coordinates. ν denotes the physical noise; the value would follow the Gaussian distribution.

Thus, the GP is defined as the function of the mean and covariance, which represent the principal characteristic and structure of the model. In addition, the RSS value in location positioning can be derived to the absolute distance; in our study, the authors derived the log-distance path-loss model as the mean function; that is:

(5) $$m\left(AP_{*}\right)=\frac{\sum_{j=1}^{N}\alpha_{j}\times \left(C_{j}-\zeta_{j}\times log\left(||AP_{*,j}-AP_{*,j}^{S}||\right)+\varepsilon_{j}\right)}{\sum_{j=1}^{N}\alpha_{j}}$$

where $C_{j}$ is a constant value, $AP_{*,j}$ is the collected online RSS value of unknown position * at *j*th AP, and $AP_{*,j}^{S}$ is the RSS value of *j*th AP at the source of unknown position *. $\alpha_{j}$ represents the weight of $AP_{*,j}$, which indicates the trustable degree of the AP. $\zeta_{j}$ represents the path-loss exponent of *j*th AP, which is computed as the noise degree of *j*th AP. In our method, this value follows the Gaussian distribution $N\left(0,\sigma_{i}^{2}\right)$; $\sigma^{i}\;$is the covariance of noises in our experiment. Accordingly, the covariance function is defined as follows:

(6) $$k\left(AP_{*},AP_{r}\right)=exp\left(\frac{\sum_{j=1}^{N}\beta_{j}||AP_{*,j}-AP_{j}||}{\sum_{j=1}^{N}\beta_{j}}\right)$$

where $\beta_{j}$ is computed as the weight of $AP_{j}$. To reduce the computation complexity, we derive the mod function; that is, we use $AP_{r}^{m}$ instead of $AP_{r}$ in Equation (6).

The objective of our system is to predict the RSS value as $f_{n}\left(AP_{*}\right)$ for all two-dimensional inputs x and y. To achieve this, the system models are defined as:

$$f_{n}\left(AP_{*}\right)\sim GP\left(m\left(AP_{*}\right),k\left(AP_{*},AP_{r}\right)\right)$$

That is, the GP models for the indoor positioning are divided into parts: one part is based on the *x* and *y* coordinates of all training locations. Thus, we let $X_{n}≜\left[x_{1},x_{2},\ldots ,x_{S}\right]$ be a set of training samples, where *S* represents the number of training coordinate data; then:

$$S_{x}=\left\{\left(x_{1},\;y_{n}\left(x_{1}\right)\right),\left(x_{2},\;y_{n}\left(x_{2}\right)\right),\;\ldots \left(x_{S},\;y_{n}\left(x_{S}\right)\right)\right\}$$

and:

$$S_{y}=\left\{\left(x_{n}\left(y_{1}\right),\;y_{1}\right),\left(x_{n}\left(y_{2}\right),\;y_{2}\right),\;\ldots \left(x_{n}\left(y_{S}\right),\;y_{S}\right)\right\}$$

are the *S*-dimensional column vectors containing the RSS measurements from all training locations.

Accordingly, we define the GP model to predict location as follows:

(7) fx(AP*)=∑j=1Nαj×(Cj−ζj×log(||AP*,j−AP*,jS||)+εj)∑j=1Nαjfx(AP*)=∑j=1N+exp(∑j=1Nβj||AP*,j−APj||∑j=1Nβj):xϵ{1,2,⋯,w}

and:

(8) fy(AP*)=∑j=1Nαj×(Cj−ζj×log(||AP*,j−AP*,jS||)+εj)∑j=1Nαjfx(AP*)=∑j=1N+exp(∑j=1Nβj||AP*,j−APj||∑j=1Nβj):yϵ{1,2,⋯,h}

We use the hyper parameter vector θ≜[C,α,ε,β] to optimize our GP model; different parameters would influence the estimated results. In our experiments, we trained hyper parameters by training the data $S_{x}$ and $S_{y}$.

The GPR has many kernels, some of which are listed in Table 1 and Table 2. The performance of the GPR was validated using the different sets of mean and covariance kernel functions. The variances were examined in our experiments.

**Figure 3:**
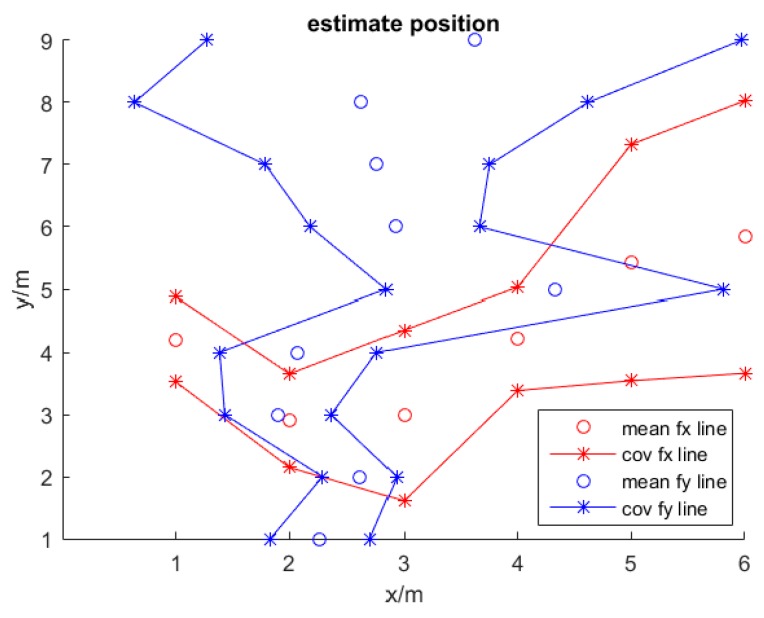
The demonstration of position predicting with GPR, red line is computed with the fixed *y* coordinate, blue line is computed with the fixed *x* coordinate. The results reveal the barriers of the upper and lower covariance values.

Figure 3 presents the results from the GPR model of Equations (7) and (8); the red line reflects the GPR model with six values of $S_{y}$, which was the location predicted by the model with the fixed y coordinate. These two red start lines indicated the barriers of the upper and lower covariance values, one cycles red line tracked the change path of the mean values. Besides, the blue line reflects the GPR model with $S_{x}$, and with the fixed *x* coordinate. Two blue start lines and one cycles blue line are represented the same things as red lines. This figure reveals that possible predicting results are barrier inside the covariance lines, and the *x* and *y* locations with those derived positions are intersected inside these areas.

### 3.2. Naive Bayesian Location Model

Our proposed system derived the positions *x* and *y* with $S_{x}$ and $S_{y}$ individually, sometimes, $S_{x}$ and $S_{y}$ have multiple solutions. To combine these results, our system employed the Naive Bayesian model. Bayes’ theorem is a simple mathematical formula used for calculating conditional probabilities. If E is a random experiment and $B,A_{1},A_{2},\cdots ,A_{n}$ are events in E, then the following conditions are met:

(1) $P\left(A_{i}\right)>0,\;i=1,2,\cdots ,n$,
(2) Events $A_{1},A_{2},\cdots ,A_{n}$ are partitioned by the sample space; their values are independent of each other,
(3) P(B)>0.

Therefore, $P\left(A_{i}|B\right)=\frac{P\left(A_{i}\times B\right)}{P\left(B\right)}=\frac{P\left(A_{i}\right)P\left(B|A_{i}\right)}{\sum_{j=1}^{n}P\left(A_{i}\right)P\left(B|A_{j}\right)},\;i=1,2,\cdots ,n$. This equation can be proved using the conditional probability theorem and total probability theorem [27].

Bayesian Decision Theory is built on Bayesian probabilities, which enable us to assert a prior belief in a data point coming from a certain class [28]. This theory is used as a classifier such as the Naive Bayesian classifier, which can classify rapidly and accurately [29,30]. According to our proposed method, RSS values transmitted from APs were all independent, and our proposed GPR model derived some predicted candidate sets of $S_{x}$ and $S_{y}$. To reduce the possible sets, the Naive Bayesian positioning method was used to combine and derive the most satisfactory results. The Naive Bayesian positioning method is detailed as follows:

(1) The sample space is divided by $S_{x}$ and $S_{y}$, and events in the sample spaces are represented as $L_{i}=\left(x_{i},y_{i}\right)$, which is the place derived from the proposed GPR method.
(2) The prior probability $P\left(L_{i}\right)$ is computed as:
  (9) $$P\left(AP_{*}|L_{i}\right)=P\left(AP_{1},AP_{2},\cdots ,AP_{n}|L_{i}\right)=\overset{n}{\underset{j=1}{\sum }}P\left(AP_{j}|L_{i}\right)\;$$
(3) The posterior probability is computed as:
  (10) $$P\left(L_{i}|\;AP_{*}\right)=\frac{P\left(AP_{*}|L_{i}\right)}{P\left(AP_{*}\right)}=\frac{P\left(AP_{*}|L_{i}\right)P\left(L_{i}\right)}{\sum_{j=1}^{n}P\left(AP_{*}|L_{j}\right)P\left(L_{j}\right)}\;$$

Accordingly, the final estimation location is defined as:

$$L_{i}:P\left(L_{i}|\;AP_{*}\right)=\text{max}\left(P\left(L_{i}|\;AP_{*}\right)\right)$$

and if $P\left(L_{i}|\;AP_{*}\right)$ is equal to zero, x and y are selected as follows:

(11) $$P\left(AP_{*}|x_{i}\right)=P\left(AP_{1},AP_{1},\cdots ,AP_{n}|x_{i}\right)=\prod_{j=1}^{n}P\left(AP_{j}|x_{i}\right)$$

and:

(12) $$P\left(x_{i}|AP_{*}\right)=\frac{P\left(AP_{*}|x_{i}\right)}{P\left(AP_{*}\right)}=\frac{P\left(AP_{*}|x_{i}\right)P\left(x_{i}\right)}{\sum_{j=1}^{n}P\left(AP_{*}|x_{j}\right)P\left(x_{j}\right)}\;$$

then:

(13) $$P\left(y_{i}|AP_{*}\right)=\frac{P\left(AP_{*}|y_{i}\right)}{P\left(AP_{*}\right)}=\frac{P\left(AP_{*}|y\right)P\left(y_{i}\right)}{\sum_{j=1}^{n}P\left(AP_{*}|y_{j}\right)P\left(y_{j}\right)}$$

(4) The final location and error between the estimate positions are computed as follows:
  $$P\left(x_{i}|AP_{*}\right)=\text{max}\left(P\left(x_{i}|\;AP_{*}\right)\right),P\left(y_{i}|AP_{*}\right)=\text{max}\left(P\left(y_{i}|\;AP_{*}\right)\right)$$
  and:
  $$P\left(y_{i}|AP_{*}\right)=\text{max}\left(P\left(y_{i}|\;AP_{*}\right)\right),P\left(x_{i}|AP_{*}\right)=\text{max}\left(P\left(x_{i}|\;AP_{*}\right)\right)$$

We then define the real location as $TL_{i}$ and ${\text{FL}}_{i}=\left\{\left(x_{i},y_{i}\right)\right\}$, and the RMSE is derived as:

(14) $$\text{Error}=\sqrt[{2}]{\frac{\sum_{i=1}^{N}{\left(TL_{i}-FL_{i}\right)}^{2}}{N}}$$

## 4. Experiments and Discussion

### 4.1. Experiment Environment Initialization

In this section, we conducted experiments in two environments. One involved simulated experiments programmed using Matlab 2015. The other environment was the physical experiment, the area of which included four beacons deployed on the corner of a square area that was 30 m × 30 m. In the simulation experiment, the signal was smooth; thus, the RSS energy declined gradually from the source; thus, the received energy could be predicted easily according to the distance from the source. However, the physical experiment was performed on the second floor in the 55th office building of Tianjin University. It was approximately a 7 m × 15 m area (Figure 3). The area was a typical building hall with some pillars in each corner of the area and was relatively open in the center, with people able to walk around freely. Four Bluetooth transmitters were placed in the corner of the area to ensure the signal full coverage of the testing area. 

**Figure 4:**
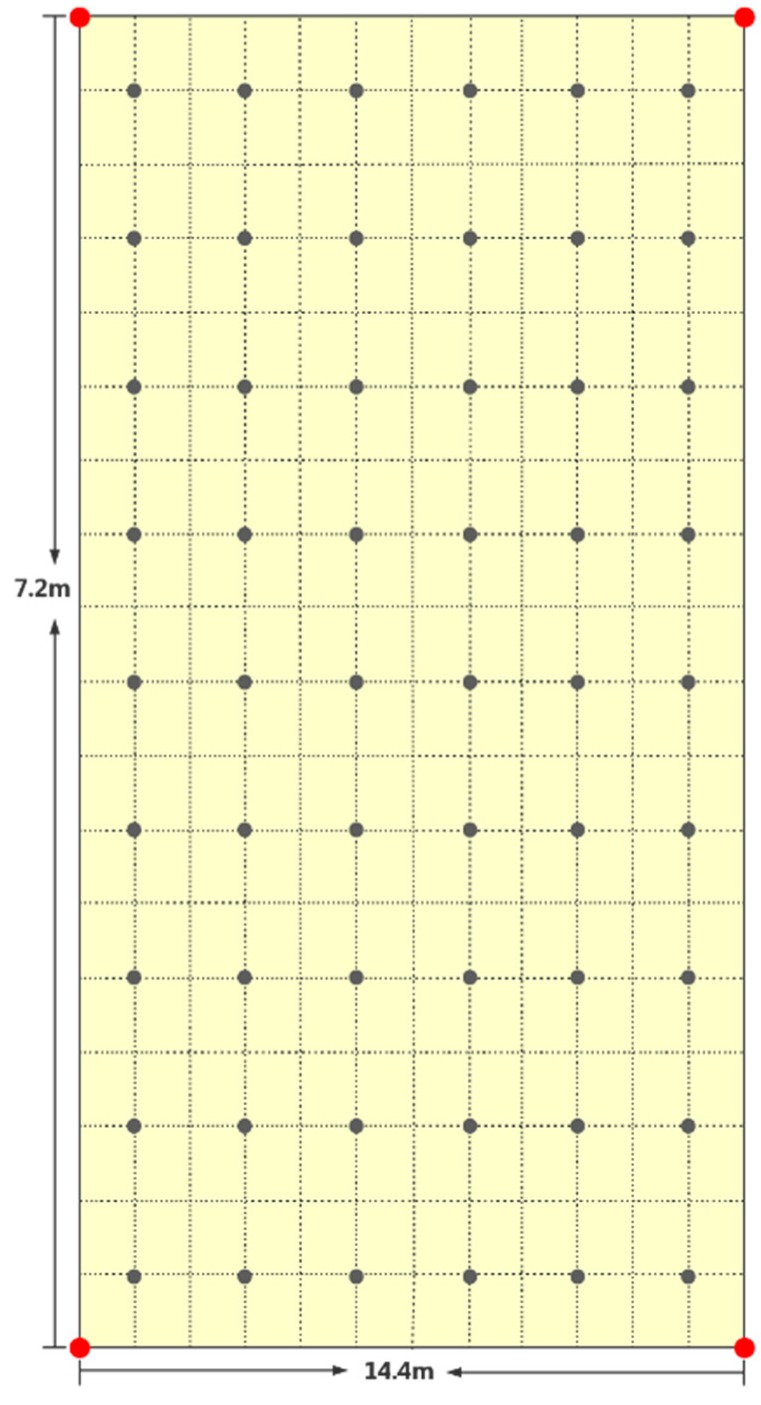
Physical testing area.

The testing area was divided into 54 locations for area measurements. The locations are marked by black dots in Figure 4. The four Bluetooth transmitters are marked by the red dots. For receiving online RSS data, we employed a Samsung Note 3 as the data collector in the testing area. Each Bluetooth transmitter was designed to transmit 100 signals per second, and our system collected 20 samples in each location; 15 signals were for system training, and five signals were for localization testing. In addition, we applied our GPRP method with the mod function to avoid potential overfitting problems through a single signal measurement.

### 4.2. The Effect of the Different Training Data Size

**Figure 5:**
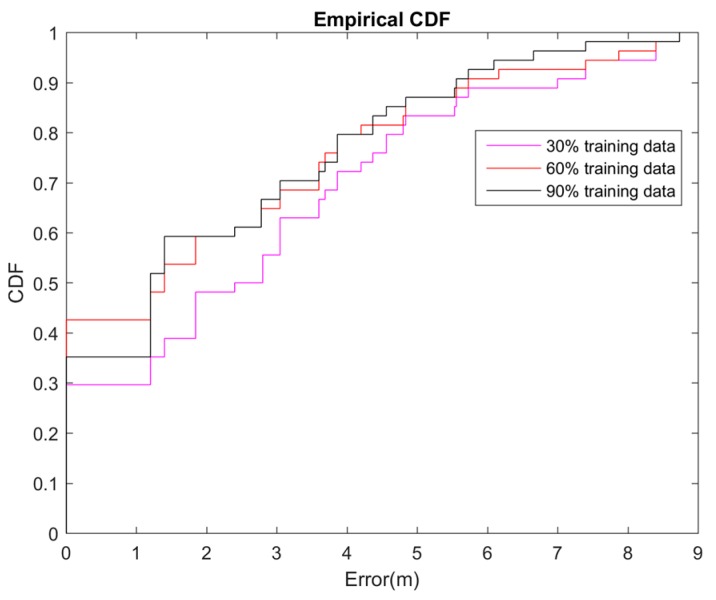
CDF of localization error for different training dataset sizes.

For the purpose of reducing the burden of the RSS data collection in the offline phase in this experiment, the authors attempted to find a balance between using limited training data and increasing the localization accuracy in the emulation environment. Figure 5 shows the cumulative distribution function (CDF) of the localization error for different training dataset sizes. We tested distinct 30%, 60%, and 90% data sizes to evaluate the performance of our proposed method. The results showed that larger training datasets achieve more satisfactory results than do smaller training datasets. The average error rates were 2.98, 2.69, and 2.45 m for the 30%, 60%, and 90% size of datasets, respectively (Figure 5). Comparing to the 2.41 m error rate of whole dataset, we can tell that the performance is acceptable at the saving almost half training time, but only increasing 0.11 times of the error rate.

### 4.3. Effect on Different GPR Kernel

**Figure 6:**
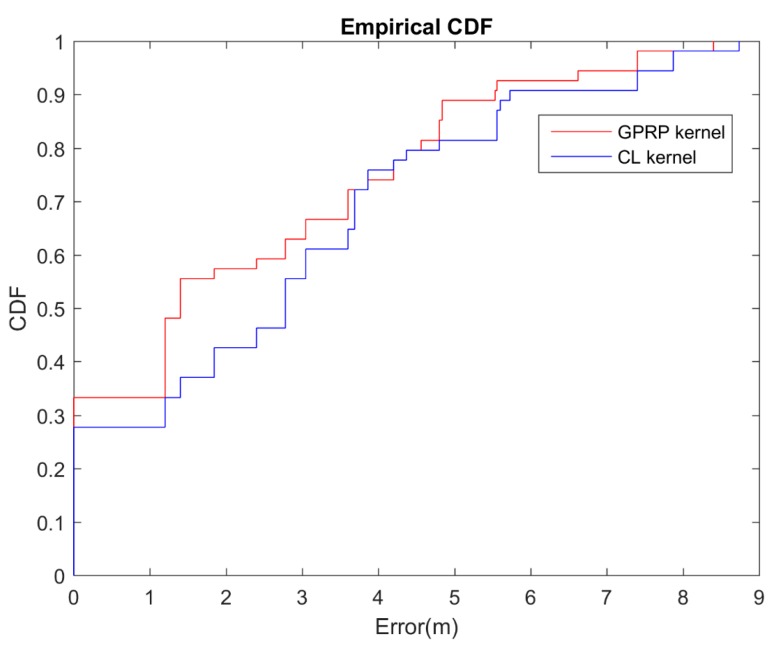
CDF of localization error for different kernels comparison.

To test location estimation accuracy with different GPR kernels, 30% of the 54 reference locations in the training area (black dots in Figure 2) were selected in this experiment; resets were used for the testing. The GPR was the kernel we adopted in our proposed GPRP method described in Section 3.1. In addition, we compared the derived results with different GPR kernels, it is the CL kernel, the CL kernel was found to be in Section 3.1 too. The CL kernel is based on the constant mean and linear covariance kernel; Table 1 and Table 2 show the equation. The popular reason of using CL kernel is because of the simplified computation. The results show that our proposed GPRP method outperformed the CL kernel (Figure 6). Table 3 summarizes the overall results, which reveal that the estimation accuracy of the GPRP method was acceptable, especially inside the 4-m area. The average computation times were 42.80 and 43.81 s for the CL and our proposed method, respectively. The accumulative accuracy of our proposed model was much higher than that of the CL method, though the computation time was only increased by approximately 2%.

### 4.4. Performance Evaluation of GPRP

To check the obstacle effects on the signal transmission in these simulations, we tested the signal transmission on several situations; one situation was tested using the same environment with different obstacles, and the other situation was tested robustly by the different obstacles.

#### 4.4.1. GPRP Robustness on Location Accuracy Testing

**Figure 7:**
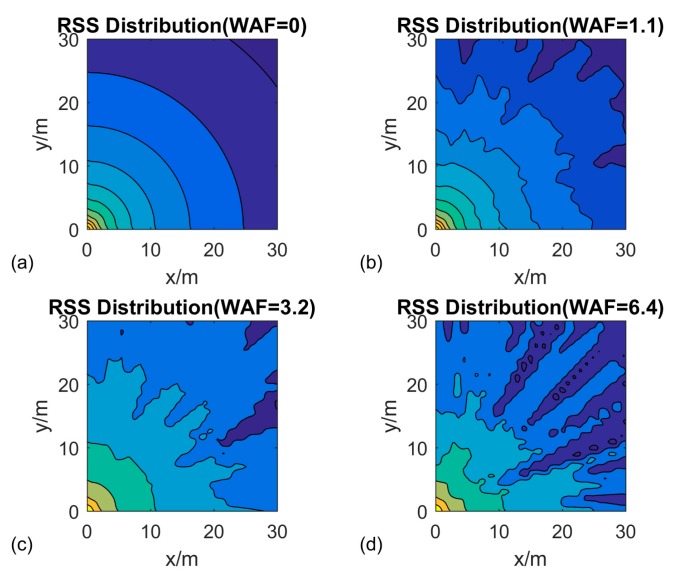
RSS testing with different obstacles.

According to Equation (1), the WAF is defined as the obstacle factor, which is used to represent the obstacle effects on the RSS. Figure 7a shows the optimal situation with receiving signals; the distribution is displayed as the cyclic shape. In addition, Figure 7b–d indicate strength mapping, with WAF of 1.1, 3.2, and 6.4, respectively These values were indicated as light, medium, and heavy crowds in the environment. According to these pictures, we can observe that the WAF affects the simulation environment.

**Figure 8:**
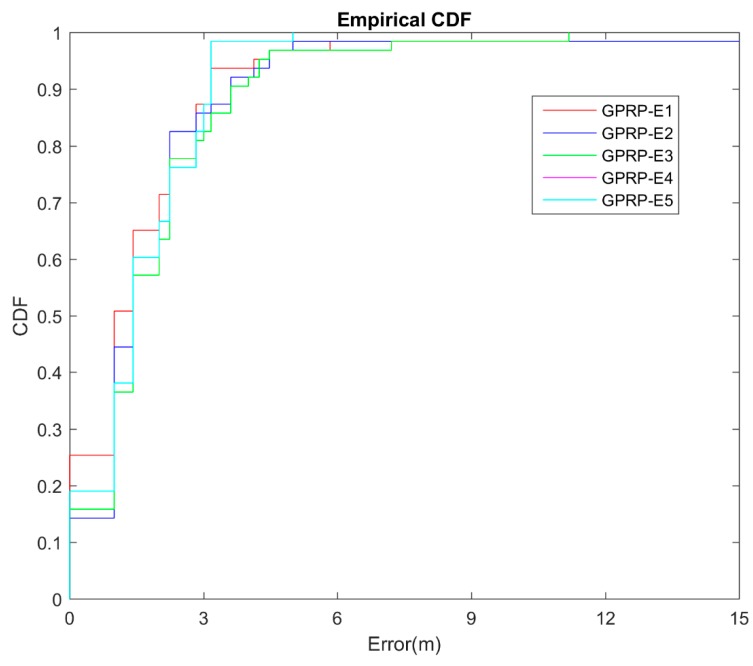
CDF of localization error for different obstacle environments.

To test the robustness, the validation was performed according to the changing environment. Thus, the WAF value was changed on the offline training and online testing. We defined four different environments in Table 4. These four environments were represented by the same offline training and dynamic online testing with light, medium, heavy, and super heavy crowds. Figure 8 shows that the GPRP method could adjust to the dynamic environments in terms of acceptable accuracy. For example, we can observe that the accuracy decrease to less than 10%, even in the light-crowd training to heavy-crowd testing.

### 4.5. Localization Accuracy on Different Environments

In these experiments, we evaluated our system performance in two different environments: simulated and field. In the environment of the field experiment, the location examined was the second floor of the 55th building of Tianjin University. For all the experiments, we adopted 30% data for training and 70% data for testing. Firstly, we compared three methods for the general fingerprinting database: our proposed GPRP, the method of Yiu [12], general fingerprinting (FP), and Weight-RSS by Zheng et al. [8]. The FP and Zheng methods are all based on the RSS location system, which is required to build an RSS mapping database of an entire area. Both the computation time and the database preparation are bottlenecks in the system execution.

Accordingly, we employed our proposed method in the actual environment. The area was divided into 6 × 9 blocks. Every block was approximately 1.4 m long and 1.2 m wide. All the experiments were carried out by using 30% data for training and 70% data for training. About the FP and Zheng method, we need to build the fingerprint database, it took 54 × 10 s = 540 s to accomplish, FP method took another 1 s to predict the online location, and Zheng method took more time (2 s) to predict. Furthermore, Yiu method and our proposed method were selected 0.3 percent samples to build the training database, the computation time was 54 × 0.3 × 10 s = 160 s to construct the basement. However, according to our description in Section 2, Yiu’s method cost average 25 s to train the GPR model, 75 s to train parameters in the model, and 46 s to rebuild the fingerprinting database on the average. Overall, Yiu’s method would spend about 307 s to finish the prediction. Besides, when our proposed method built the fingerprint database, we only needed 25 s to train the GPRP model, after that, the offline prediction would cost about 125 s to complete this procedure. Overall, our proposed method only cost 205 s to predict the online position on the average. Table 5 showed all the data for these four methods. According to the comparing results in the Table 5, the results reveal that the computation time would be extended with the sampling size of fingerprint database, because the procedure of RSS value re-mapping to the position was changeable. Our proposed method not only can save the computation time of building the RSS fingerprint database, but also can remain the accuracy of online prediction.

**Figure 9:**
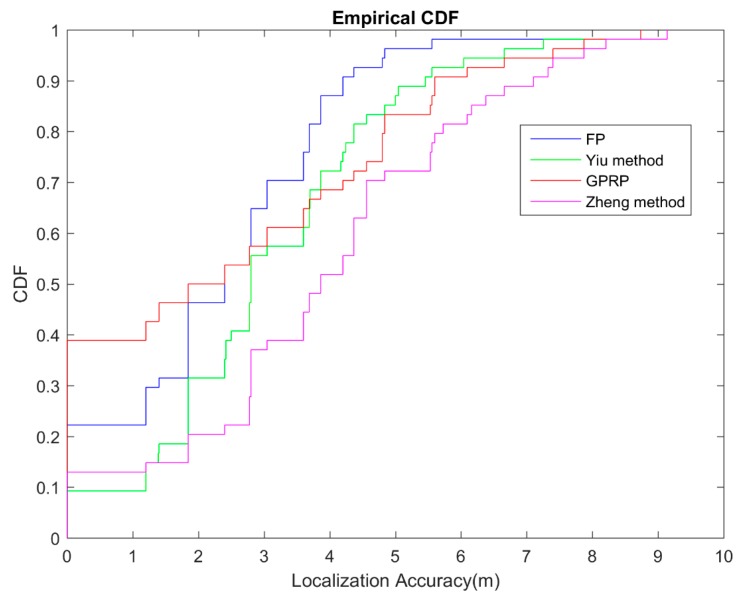
CDF of physical localization error for different algorithms comparison.

In this experiment of physical area, we compared four methods for the general fingerprinting database: our method GPRP, the method of Yiu, the method of Zheng, and fingerprinting (FP). The graph (Figure 9) below showed all the comparison. The average errors were 2.28, 2.34, 3.09 and 2.93 m for our proposed GPRP method, the FP method, the Yiu method, and Zheng method respectively. The combination of graph and table showed that our proposed method can retain accuracy and reduce computation time in the simulated localization system.

**Figure 10:**
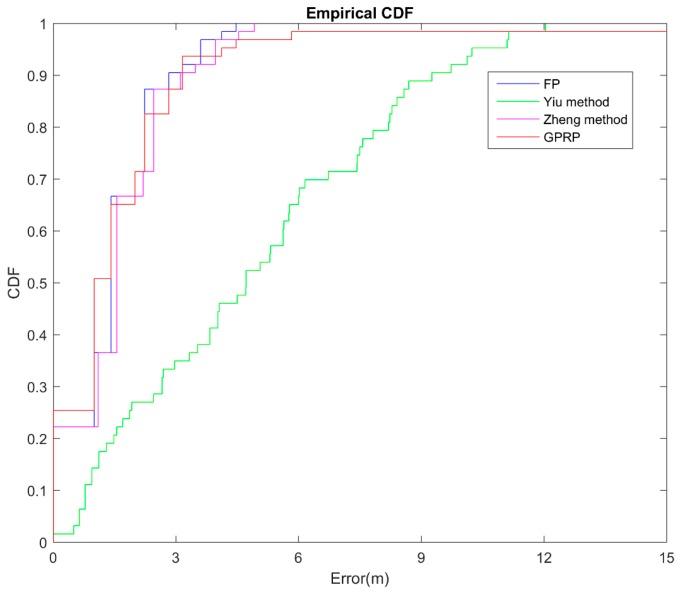
CDF of simulated localization error for different algorithms comparison.

In the simulation area, when we compared our method to the FP method, the results showed that the performance of our method was almost the same as that of the FP method (Figure 10); however, our method required only half the computation time of the FP method. This advantage was based on the saving of comparing process from the lookup table. In addition, the Yiu method had the partial advantage of flexibility, though it had the longest computation time of all three methods. The average errors were 1.82, 1.46, 4.88, 7.2 and 1.6 m for our proposed GPRP method, the FP method, the Yiu method, and Zheng method respectively. The cumulative error rate at 3 m was 0.87, 0.90, 0.34 and 0.85 for the GPRP method, the FP method, the Yiu method and Zheng method, respectively. Overall, the current results still revealed that our proposed method can retain accuracy and reduce computation time in the simulated localization system.

## 5. Conclusions

Generally, the main challenge in RSS-based location positioning is the high sensitivity of the technique to environmental changes. Variations in RSS measurement reduce estimation accuracy. In other words, if the radio propagation signal strength were correlated with the distance between the transmitter and the receiver, location determination would be a trivial problem. However, the relationship between these two parameters is dynamic rather than straightforward. Hence, many methods have been proposed for obtaining location predictions when the position of the receiver is changing. One such basic method is using a fingerprinting database, which involves straight computation for RSS signal collection. Both time consumption and searching from the lookup table database are bottlenecks in this method.

In this study, we proposed the GPRP method for adapting the flexibility of the GP and determining the location by using the simplified computation of the Naive Bayes method. Both computation time and efficiency can be improved using this method. In our experiments, we tested our method by examining the 55th building of Tianjin University in both a simulated and physical environment. The experiments considered distinct data size, different kernels, and accuracy testing. The results showed that our proposed method can ensure accuracy and reduce computation time in location prediction.

## Figures and Tables

**Table 1:** Summary of several commonly-used mean functions [26].

Mean Function	Equation Expression
Zero	0
Constant	∁; ${\left(x\times x^{\prime }+\sigma_{0}^{2}\right)}^{p}$
Linear	αx+b
Poly	$\sum_{i}^{N}\alpha^{i}x^{N-i}$

**Table 2:** Summary of several commonly-used covariance functions [26].

Covariance Function	Equation Expression
Constant	$\sigma_{0}^{2}$
Linear	$\sum_{d=1}^{D}\sigma_{d}^{2}x_{d}x_{d}^{\prime }$; ${\left(x\times x^{\prime }+\sigma_{0}^{2}\right)}^{p}$
Polynomial	exp(−r^2/(2l^2 ))
Exponential	exp(−r/l)
Rational quadratic	(1+r^2/(2αl^2 ))^(−α)

**Table 3:** Localization results in testing area.

	RMSE (m)	1 m	2 m	3 m	6 m
**GPRP method**	2.28	33.33%	57.41%	62.96%	92.59%
**CL method**	2.78	27.79%	42.59%	55.56%	90.74%

**Table 4:** Robustness testing for four different environment.

ID for Testing Environment	WAF for Off-Line Training	WAF for On-Line Testing
E1	0.2	0.2
E2	0.2	0.3
E3	0.2	0.5
E4	0.2	1
E5	0.2	2

**Table 5:** Total performance of four comparison methods.

	Simulated Experiments		Field Experiments	
	Time Cost (s)	Average Error (m)	Time Cost (s)	Average Error (m)
FingerPrint (FP)	4201	1.46	541	2.34
Yiu method [12]	1385	4.88	307	3.09
Zheng method [8]	4203	1.6	542	2.93
GPRP	1395	1.82	205	2.28
